# Antinociceptive Activity of the Ethanolic Extract, Fractions, and Aggregatin D Isolated from *Sinningia aggregata* Tubers

**DOI:** 10.1371/journal.pone.0117501

**Published:** 2015-02-26

**Authors:** Geórgea V. Souza, Alex S. Simas, Amanda L. Bastos-Pereira, Gisele R. A. Frois, João L. C. Ribas, Maria H. Verdan, Cândida A. L. Kassuya, Maria E. Stefanello, Aleksander R. Zampronio

**Affiliations:** 1 Department of Pharmacology, Federal University of Paraná, Centro Politécnico, PO Box 19031, Curitiba, PR, 81531-980, Brazil; 2 Department of Chemistry, Federal University of Paraná, Centro Politécnico, PO Box 19081, Curitiba, PR, 81530-900, Brazil; Xi'an Jiaotong University School of Medicine, CHINA

## Abstract

The present study investigated the effects of the ethanolic extract (ESa), fractions, and compounds isolated from *Sinningia aggregata* in male Swiss mice on carrageenan-induced paw edema, neutrophil migration, mechanical hyperalgesia, formalin-induced nociception, and lipopolysaccharide-induced fever. The ESa did not alter edema, neutrophil migration, or fever at any of the doses tested. However, the ESa reduced phase II of formalin-induced nociception and carrageenan-induced mechanical hyperalgesia. The petroleum ether (PE) and ethyl acetate (EA) fractions and aggregatin D (AgD; isolated from the EA fraction) reduced formalin-induced nociception. Anthraquinones from the PE fraction were ineffective. AgD also inhibited carrageenan-induced mechanical hyperalgesia. Neither the ESa nor AgD altered thermal nociception or motor performance. Local administration of AgD also reduced hyperalgesia induced by carrageenan, bradykinin, tumor necrosis factor-α, interleukin-1β, cytokine-induced neutrophil chemoattractant, prostaglandin E_2_, and dopamine but not hyperalgesia induced by forskolin or dibutyryl cyclic adenosine monophosphate. The positive control dipyrone reduced the response induced by all of the stimuli. Additionally, glibenclamide abolished the analgesic effect of dipyrone but not the one induced by AgD. AgD did not change lipopolysaccharide-induced nitric oxide production by macrophages or the nociception induced by capsaicin, cinnamaldehyde, acidified saline, or menthol. These results suggest that the ESa has important antinociceptive activity, and this activity results at least partially from the presence of AgD. AgD reduced mechanical hyperalgesia induced by several inflammatory mediators through mechanisms that are different from classic analgesic drugs.

## Introduction

Pain is one of the most pervasive problems in our society and has high social and economic impacts [1]. During inflammation, several mediators can activate and/or sensitize nociceptive fibers such as bradykinin (BK) [2], substance P (SP) [3], cytokines (e.g., tumor necrosis factor-α [TNF-α] and interleukin-1β [IL-1β]), prostaglandins and sympathetic amines [4]. In addition to pain, similar mediators are involved in edema formation and leukocyte infiltration [5–9]. If some mediators, particularly cytokines, reach the circulation, then they can cause fever by its actions in areas near the hypothalamus [10]. Several analgesics are used to treat a wide range of painful and inflammatory conditions including non-steroidal anti-inflammatory drugs (NSAIDs) [11], glucocorticoids [12] and opioids [13]. Aside from these drugs, other drugs have been used for specific painful conditions [14, 15]. Despite the great diversity of available antiinflammatory and analgesic drugs, their side effects and the ineffectiveness of some drugs in some conditions require the continuous search for new drugs.

The genus *Sinningia* belongs to the Gesneriaceae family and comprises 68 species that are distributed in South America. Many of them are found in Brazil [16]. The chemical composition of *Sinningia* species has been studied in the last few years. Flavonoids were isolated from *S*. *cardinalis* [17], and ethylcyclohexane derivatives and anthraquinones were identified in *S*. *speciosa* [18]. In *S*. *allagophylla*, lapachenol, 8-methoxylapachenol, anthraquinones, and naphthoquinones were found [19]. *S*. *aggregata* produces essential oil, anthraquinones, and aromatic compounds with a new skeleton named aggregatin A-D [20, 21].

Although the chemical composition of these plants is beginning to be known, few studies have investigated the pharmacological properties of the newly identified compounds. We recently found that an ethanolic extract from *S*. *allagophylla* exerted antinociceptive effects, an action related, at least partially, to the presence of 8-methoxylapachenol [22]. Anthraquinones were also found in these species, a class of compounds usually associated with antiinflammatory and antinociceptive activity in other species [23–25]. These observations prompted us to investigate the possible antinociceptive, antiinflammatory and antipyretic activity of the ethanolic extract obtained from the tuber of *S*. *aggregata* (ESa). Once the antinociceptive activity of the ESa was identified, we further investigated the activity of the fractions and isolated compounds obtained from the ESa. We identified antinociceptive effects in one of these compounds, aggregatin D (AgD), and evaluated its effectiveness against nociception induced by several mediators and ion channels agonists, and nitric oxide (NO) production to obtain some indications about its mechanism of action.

## Materials and Methods

### Animals

The experiments were conducted using male Swiss mice (25–35 g), that were housed 5 per cage containing sterile wood shavings under a 12h light/dark cycle, with controlled humidity (60–80%) and temperature (22 ± 1°C). Mice from the Biologycal Sciences Section standard breeding unit from the Federal University of Paraná were used and food and water were freely available. Animals were acclimatized to the experimental room at least 2 h before testing and were used only once throughout the experiments. The studies were performed in accordance with the current Brazilian (Conselho Nacional para o Controle de Experimentação Animal) and International guidelines for the care of laboratory animals. The animal procedures were approved by the Institutional Animal Care and Use Committee (Comissão de Ética para o Uso de Animais, Setor de Ciências Biológicas, Universidade Federal do Paraná CEUA/BIO-UFPR, authorization no. 628 and 722). The number of animals used was the minimum number necessary to show consistent effects of the drug treatments. All efforts were made to minimize animal suffering. At the end of the experiments, the animals were anesthetized with 60 mg/kg ketamine plus 7.5 mg/kg xylazine and euthanized by cervical dislocation.

### Plant material

*Sinningia aggregata* (Ker-Gawl.) Whiehler (tubers) was collected in Tibagi, Paraná, Brazil, (24°30′32″ S, 50° 24′ 50″ W) in May 2007 and identified by Clarice B. Poliquesi. A voucher specimen (no. 290738) was deposited in the herbarium of Museu Botânico Municipal in Curitiba, Paraná, Brazil. *S*. *aggregata* is not an endangered species or protected, and authorization is not required for its collection. The authorization for accessing and studing samples from the Brazilian genetic heritage was obtained from the Brazilian governament through Conselho Nacional de Desenvolvimento Ciêntífico e Tecnológico (CNPq authorization no. 010087/2012-5).

### Extraction and isolation

**Fig 1:**
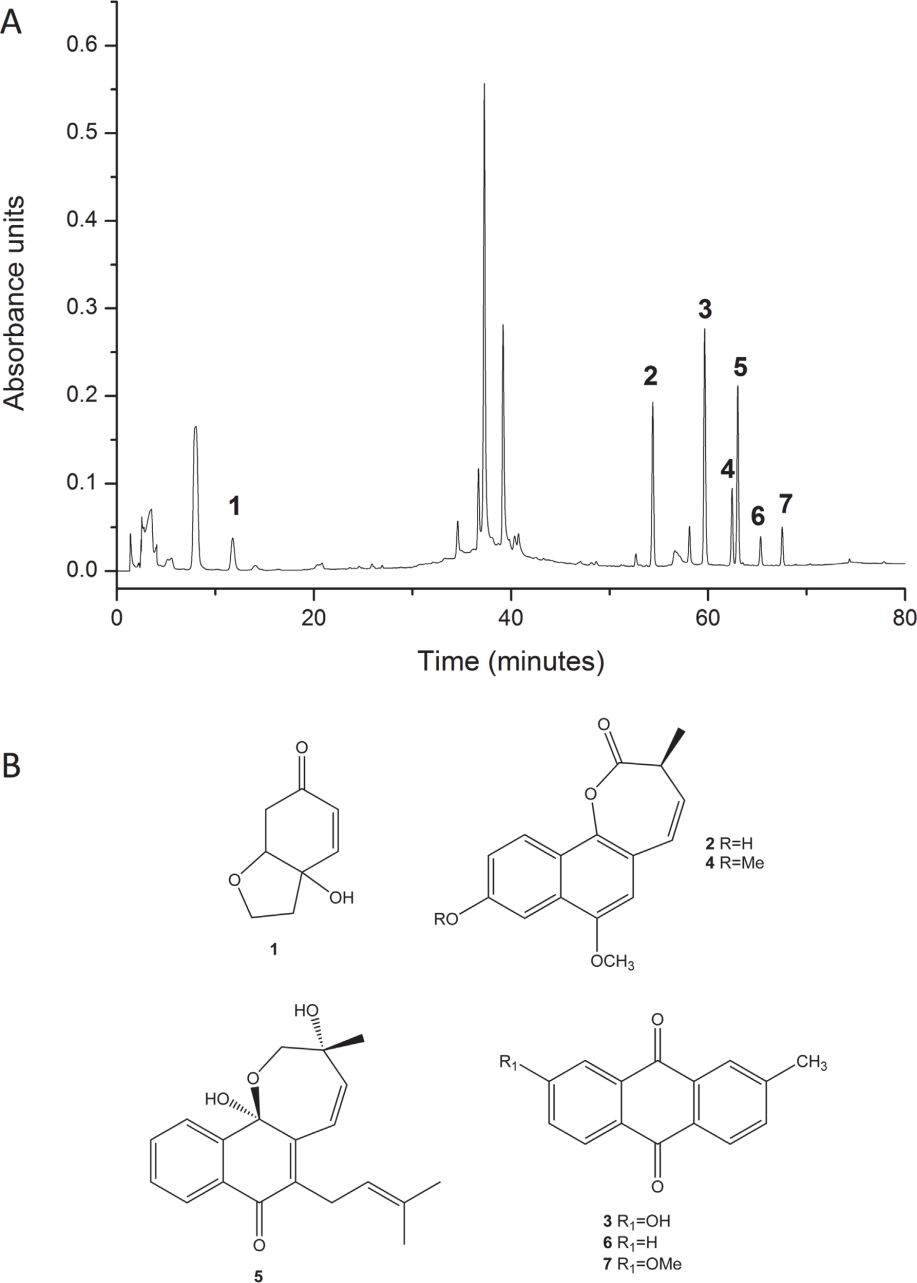
Fingerprint of the ESa obtained from the tubers of *S*. *aggregata*. The structures of the compounds identified are shown in panel B and are as follows: halleridone (**1**), aggregatin A (**2**), 7-hydroxy-2-methylanthraquinone (**3**), aggregatin C (**4**), aggregatin D (**5**), 2-methylanthraquinone (**6**) and 7-methoxy-2-methylanthraquinone (**7**).

Dried and powdered tubers (50.5 g) were extracted at room temperature with ethanol (3 × 200 ml) and the solvent was removed under reduced pressure to yield the crude ESa (4.2 g). The crude ESa was suspended in ethanol:water 1:1 and subjected to partition with petroleum ether (PE), dichloromethane (DM), ethyl acetate (EA), and 1-butanol. After solvent removal the fractions yielded 4.1%, 5.0%, 23.3% and 35.1%, respectively. Biological assays showed that the activity was concentrated in the PE and EA fractions. After chromatographic fractioning, 2-methylanthraquinone (0.5%) and 7-methoxy-2-methylanthraquinone (0.5%) were isolated from the PE fraction and AgD (3%) was isolated from the EA fraction. The compounds were identified by 1D and 2D nuclear magnetic resonance and compared with data from the literature. A fingerprint of ESa together with the chemical structures of the identified compounds obtained by high-performance liquid chromatographyis is shown in Fig. 1, the detailed procedure for which was described previously [21].

### Drugs

Carrageenan, lipopolysaccharide (LPS) *E*. *coli* 0111:B4, indomethacin, BK, prostaglandin E_2_ (PGE_2_), dopamine, forskolin, dibutyryl cyclic adenosine monophosphate (cAMP), glibenclamide, dipyrone, fentanyl, diazepam, 3-(4,5-dimethylthiazol-2-yl)-2,5-diphenyl tetrazolium bromide (MTT), ruthenium red, camphor, amiloride, capsaicin, cinnamaldehyde, and menthol were purchased from Sigma (St. Louis, MO, USA). Tumor necrosis factor- α (TNF-α), interleukin-1β (IL-1β) and cytokine-induced neutrophil chemoattractant 1 (CINC-1) were purchased from R&D Systems (Pittsburgh, PA, USA). Ketamine and xylazine were obtained from Syntec Laboratory (Cotia, SP, Brazil). Oxytetracycline hydrochloride was purchased from Pfizer (São Paulo, SP, Brazil). Dexamethasone was obtained from Química Santa Marina (Rio de Janeiro, RJ, Brazil).

### Carrageenan-induced edema

Edema was measured as described previously [26]. Briefly, the mice were orally treated with ESa (10–100 mg/kg), vehicle (1% Tween 20), or dexamethasone (1 mg/kg, positive control). After 1 h, they received a 50 μl subcutaneous injection of carrageenan (300 μg into the right paw) suspended in sterile 0.9% saline. The contralateral paw received only saline and was used as a control. Edema was measured using a digital micrometer (Great, MT-045B, Shanghai, China) and is expressed as the difference between the paw thicknesses (in μm) 1 h before and 0.5, 1, 2, and 4 h after the carrageenan injection.

### Lipopolysaccharide-induced fever

Abdominal body temperature was measured using data loggers (Subcue data loggers, Calgary, Canada) as described previously [26]. The data loggers were implanted intraperitoneally under 60 mg/kg ketamine plus 7.5 mg/kg xylazine anesthesia and aseptic conditions 1 week prior to the experiment. The animals were treated with 50 mg/kg oxytetracycline hydrochloride (i.m.) after surgery. Body temperature was continuously recorded at 15-min intervals from 2 h before until 6 h after an intraperitoneal injection of 100 μg/kg LPS. Controls were treated with the same volume of pyrogen-free saline (vehicle). The ESa (30 mg/kg) or vehicle (1% Tween 20) was administered orally 1 h before the LPS injection. During the experiment, the room temperature was kept at 30 ±1°C (i.e. a thermoneutral zone for mice) [27].

### Carrageenan-induced neutrophil migration

To investigate the effect of the ESa on neutrophil migration, the animals were treated with the ESa (10–100 mg/kg, p.o.), vehicle (1% Tween 20), or dexamethasone (1 mg/kg, positive control). One hour later, they received carrageenan (300 μg/paw) or saline. The animals were euthanized, as described above, 4 h after the carrageenan injection, and the tissues of the injected paws were removed. Samples were processed, and myeloperoxidase activity was assessed as described previously [28]. Protein content in the samples was measured using Bradford’s method. Enzymatic activity was determined by measuring the optical density (OD) at 630 nm and is expressed as OD per mg of protein.

### Formalin-induced nociception

The procedure was similar to the one described previously [29]. Briefly, the animals were placed in glass cylinders (20 cm diameter) for adaptation for 30 min. The mice were then orally treated with the ESa (10–100 mg/kg), PE fraction (1.30 mg/kg), DM fraction (1.50 mg/kg), EA fractoins (7.00 mg/kg), or the isolated compounds AgD (0.21 mg/kg), methylanthraquinone (1.25 mg/kg), and 7-methoxy-2-methylanthraquinone (1.25 mg/kg). The control animals received the same volume of vehicle (10 ml/kg, 1% Tween 20, p.o.) or indomethacin (5 mg/kg, p.o., positive control). After 1 h, the animals received an injection of 20 μl of 2.5% formalin (0.92% formaldehyde) in phosphate-buffered saline (PBS) in the right hindpaw. The time that the animals spent licking and elevating the injected paw (i.e. nociceptive behavior) was recorded in 5-min blocks for 30 min. The first 5-min block was considered as phase I of the test (i.e. the neurogenic phase) and the 15–30 min period was considered phase II (i.e. the inflammatory phase).

### Carrageenan-induced mechanical hyperalgesia

The mechanical threshold was measured by using von Frey filaments (Stoeling, Chicago, IL, USA) in the up-and-down paradigm as described previously [30, 31]. The mice were first acclimated (1 h) in individual clear Plexiglas boxes (9 x 7 x 11 cm) on an elevated wire mesh platform to allow access to the plantar surface of the hindpaws. The paw was then touched with a series of eight von Frey fileaments with logarithmic increments of force (0.008, 0.02, 0.07, 0.16, 0.4, 1.0, 2.0, and 4.0 g). The von Frey filaments were applied perpendicularly to the plantar surface with sufficient force to cause slight buckling against the paw and held in place for approximately 2–4 s. The absence of paw lifting after this time led to the use of the next filament with increased force. Paw lifting indicated a positive response and led to the use of the next weaker filament. This paradigm continued until six measurements were collected. The 50% mechanical paw withdrawal threshold was then calculated from these scores as described previously [31]. The animals were then treated with the ESa (3–30 mg/kg), the isolated compound AgD (0.21 mg/kg), or vehicle (1% Tween 20) orally or AgD (0.07–7 ng into the right or left paw) or the respective vehicle (0.1% Tween 20 in sterile saline, 20 μl) applied locally). Sixty minutes after oral treatment or 15 min after local treatment, the animals received a 20 μl injection of carrageenan (300 μg/paw) into the right hindpaw. The withdrawal response was measured again 3 h after the carrageenan injection.

In mice, the hyperalgesia induced by carrageenan induces the release of TNF-α and keratinocyte-derived chemokine (KC) which stimulate the release of IL-1β [4]. IL-1β and KC induce the release of prostaglandins but KC also induces the release of sympathetic amines. Both prostaglandins and sympathetic amines induce the synthesis of cAMP [4]. BK evokes nociception through a separate pathway [2]. To evaluate whether AgD affects any particular point of this cascade, animals were treated with AgD (7 ng/paw) or the positive control dipyrone (320 μg/paw) or vehicle (0.1% Tween 20). After 15 min they received local injections of different nociceptive stimuli: TNF-α (1 pg/paw), IL-1β (0.5 pg/paw), CINC-1 (10 pg/paw, which activates the same receptor as KC), PGE_2_ (100 ng/paw), BK (500 ng/paw), dopamine (3 μg/paw), dibutyryl cAMP (dbcAMP, 5 μg/paw), and forskolin (1 μg/paw). Mechanical hyperalgesia was measured after 3 h.

To evaluate if AgD exerts its action by activating NO/cyclic guanosine monophosphate (cGMP)/K^+^ pathway similarly to dipyrone, the animals were treated with vehicle (1% Tween 80 in sterile saline) or the K^+^ channel blocker glibenclamide (80 μg/paw). After 30 min they received AgD (7 ng/paw), the positive control dipyrone (320 μg/paw), or vehicle (0.1% Tween 20). After 15 min, the animals received a local injection of PGE_2_ (100 ng/paw). Dipyrone was included as a positive control in this series of experiments because it has been shown to reduce hyperalgesia induced by of all of these stimuli. The doses of nociceptive stimuli, dipyrone, and glibenclamide were based on previous studies [2, 4, 32–35].

### Hot-plate test

The hot-plate test was performed as described previously [26]. The temperature of the hot plate was maintained at 55 ± 1°C. Basal latency was measured, and only animals that presented a basal latency between 7–15 s were used. After 30 min, the mice were orally treated with the ESa (10, 30 or 100 mg/kg), AgD (0.21 or 2.1 mg/kg), vehicle (1% Tween 20) or with fentanyl (0.5 mg/kg, s.c., positive control). One hour after treatment with the extract, compound, and vehicle, or 15 min after treatment with fentanyl, the postdrug latency was evaluated. The cut-off time was 30 s. Hot-plate latencies were then converted to a percentage of the maximal possible effect (%MPE): %MPE = (postdrug latency—basal latency) / (cut-off time—basal latency) x 100.

### Locomotor performance

To evaluate the possible nonspecific muscle-relaxant or sedative effects of the ESa and AgD, the mice were subjected to the rotarod test (Ugo Basile, Model 7600, Varese, Italy) [26]. The animals were treated with ESa (10, 30, or 100 mg/kg), AgD (0.21 or 2.1 mg/kg), vehicle (1% Tween 20) or diazepam (5.0 mg/kg, s.c., positive control). One hour after ESa or AgD treatment or 15 min after diazepam treatment, animals were submitted to the rotarod test. The cut-off time was set at 180 s.

### Nitric oxide production by peritoneal macrophages

To evaluate wether AgD acts directly on macrophages, cells from the peritoneal cavity were obtained as described previously [36]. Briefly, the peritoneal cavity of halothane-anesthetized mice was washed with 6 ml of sterile PBS that contained 5U/ml heparin. The fluid was then centrifuged at 1000 x g, at 4°C for 10 min to separate the cells. The cells were then resuspended in RPMI 1640, pH 7.4 and washed twice. The cells (10^6^ cells/well) were then placed in a 96-well plate and left to adhere 1 h at 37°C in a 5% CO_2_ atmosphere. Non-adherent cells were washed away with RPMI 1640. The adhered cells were exposed to RPMI 1640 medium that contained AgD (4, 40, 400, and 4000 ng/ml) with or without LPS (100 ng/ml). Control monolayers were exposed only to RPMI 1640 or dexamethasone (9 μg/ml) plus LPS. Samples were collected after 4 h and kept at −20°C for NO evaluation using the Griess reaction as described previously [37]. For viability evaluation, the cells were incubated with 100 μl MTT (5 mg/ml) in RPMI 1640 medium for 24 h at 37°C in a 5% CO_2_ atmosphere. After this period, the formazan precipitate was dissolved with 10% sodium dodecyl sulfate solution that contained 1 M HCl. Absorbance was read at 550 nm after 4 h [38, 39].

### Nociception induced by TRP channel agonists

To test wether transient receptor potential V_1_ (TRPV_1_), TRPA_1_, TRPM_8_ and acid-sensing ion channel [40] channels might constitute potential specific targets for the antinociceptive actions of AgD, we tested the effects of AgD against nociceptive responses elicited by activators of each channel. Ten minutes following local treatment with AgD (7 ng/paw) or the positive controls ruthenium red (nonselective TRP antagonist, 2 nmol/paw), camphor (TRPA_1_ antagonist, 250 ng/paw), or amiloride (epithelial Na^+^ channel blocker, 3 μg/paw), the mice received a 20 μL intraplantar injection of the TRPV_1_ agonist capsaicin (0.1 nmol/paw), TRPA_1_ agonist cinnamaldehyde (10 nmol/paw), ASIC agonist acidified saline (2% acetic acid in 0.9% saline, pH 1.98, 20 μl/paw), or TRPM_8_ agonist menthol (2.4 μmol) into the ventral surface of the right paw. Each animal was then placed, immediately and alone, into a glass cylinder (20 cm diameter) that was positioned on a platform in front of a mirror to enable full view of the hindpaws. The amount of time that the animal spent licking the injected paw was recorded with a chronometer over 5 min (for capsaicin or cinnamaldehyde) or 20 min (acidified saline and menthol) and used as an index of nociceptive behavior intensity. The doses were based on previous studies [41, 42]

### Statistical analysis

The results are presented as mean ± s.e.mean. Statistical significance among groups was assessed using two-way repeated measures (oedema and fever) analysis of variance (ANOVA) or one-way ANOVA for the subsequent experiments. Significant mean effect and interactions in the ANOVAs were followed by the Bonferroni *post hoc* test. Dose-response curves in the mechanical hyperalgesia experiments were analyzed by one-way ANOVA followed by Fisher’s Least Significant Difference *post hoc* test. Values of *P* < 0.05 were considered statistically significant.

## Results

### Effects of the ESa on carrageenan-induced edema, neutrophil migration, and LPS-induced fever

**Fig 2:**
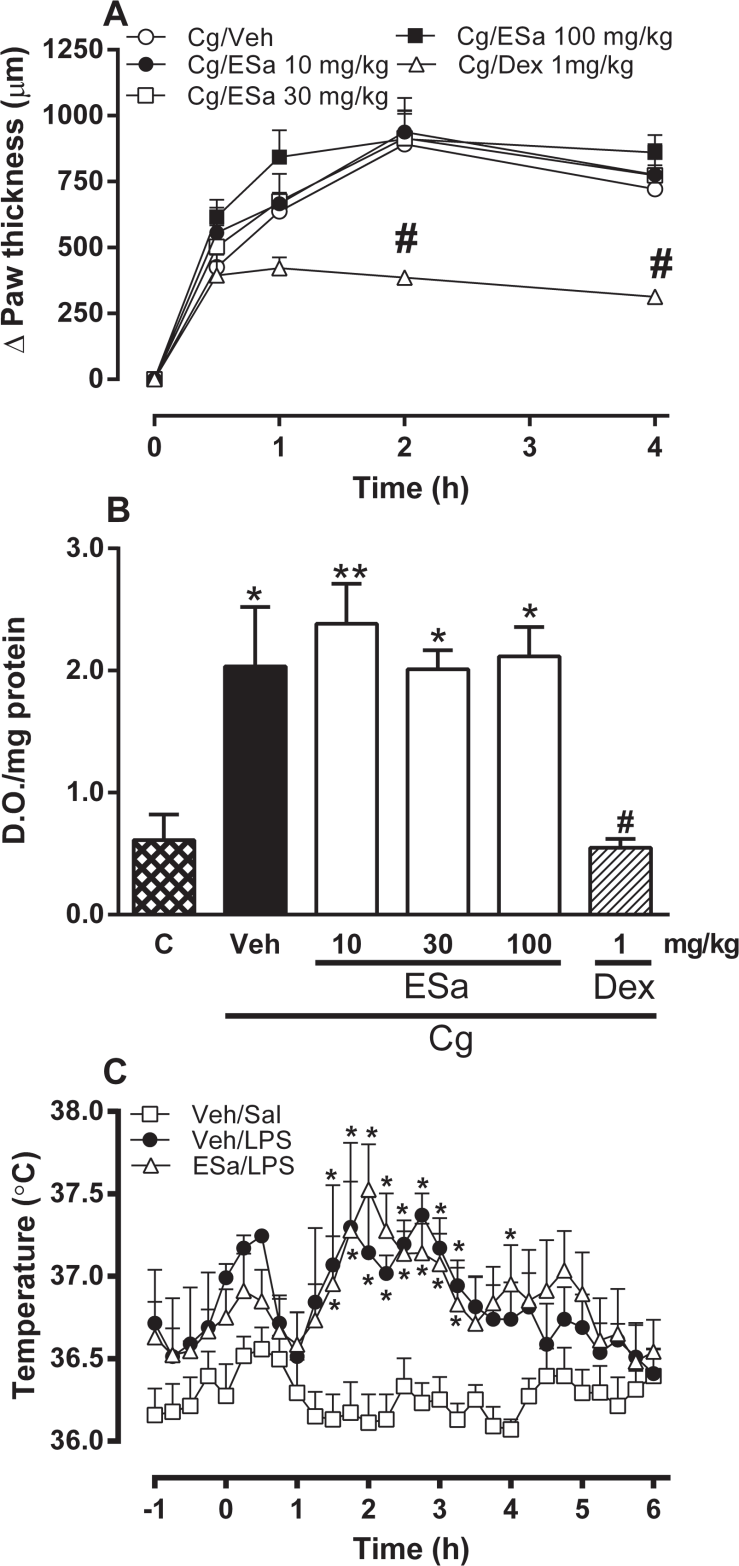
Effect of ESa on carrageenan-induced edema and neutrophil migration and on lipopolysaccharide-induced fever. Animals were treated with ethanolic extract form *S*. *aggregata* (ESa, 10 to 100 mg/kg, as indicated), Dexamethasone (Dex, 1 mg/kg), or the appropriate vehicles (Veh) by oral route. One hour after the oral treatment animals were injected with carrageenan (Cg, 300 μg) in the paw (panels A and B) or LPS (100 μg/kg, i.p, panel C). On panel B, the control (C) animals are non-injected animals. Data show the mean ± s.e.mean of the change in the paw thickness (μm, panel A), optical density (D.O./mg protein, panel B) or body temperature (°C, panel C) (n = 7–14). Symbols denote statistical difference in relation to the control (C) or Veh/Sal group (* *P*<0.05, **P<0.01) or to Veh/Cg (^#^p<0.05).

Carrageenan injection in the paw induced an edema response that began at 30 min and peaked at 2 h (Fig. 2A). Carrageenan also increased myeloperoxidase activity (an indicator of neutrophil migration) in the paw after 4 h (Fig. 2B). Oral treatment with the ESa (10–100 mg/kg) did not inhibit carrageenan-induced paw edema or myeloperoxidase activity whereas dexamethasone treatment significantly reduced both (Fig. 2A and B, respectively). The intraperitoneal injection of LPS induced a febrile response that began 1.5–2 h after the injection and persisted until 4–4.5 h. Oral treatment of the animals with the Esa (30 mg/kg) did not change LPS-induced fever (Fig. 2C).

### Effects of the ESa on formalin-induced nociception and carrageenan-induced mechanical hyperalgesia

**Fig 3:**
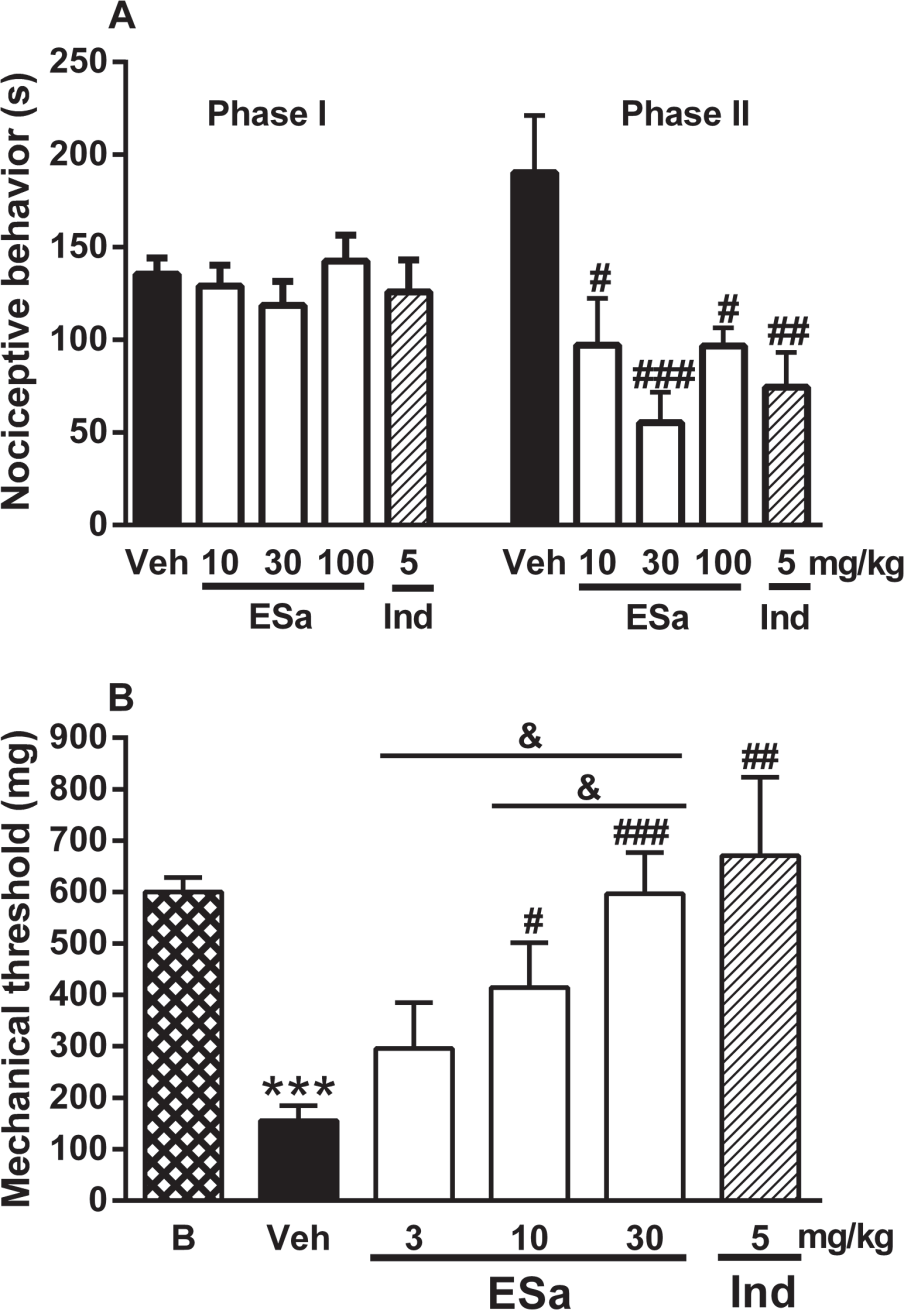
Effect of ESa on nociception. Animals were treated with ethanolic extract form *S*. *aggregata* (ESa, 10 to 100 mg/kg, as indicated), Indomethacin (Ind, 5 mg/kg) or Diazepam (Dzp, 5 mg/kg) by oral route or with Fentanil (Fent, 0.5 mg/kg, s.c.) or the appropriate vehicles (Veh) as described in Methods. One hour after the oral treatment or 15 min after s.c. tretatment, animals were injected with formalin 2.5% in the paw (panel A) or carrageenan (300 μg) in the paw (panels B) or submitted to the hot plate (panel C) or rota-rod task (panel D). On panel B, basal (B) mechanical threshold means the threshold before any injection. Data show the mean ± s.e.mean of the change in the nociceptive behavior (s, panel A), mechanical threshold (mg, panel B), MEP (%, panel C) and motor performance (s, panel D) (n = 7–14). Symbols denote statistical difference in relation to the basal (B) group (***P<0.001) or to Veh-treated animals (^#^ *P*<0.05, ^##^ *P*<0.01 and ^###^ *P*<0.001).

Formalin injection induced nociceptive behavior during phase I (approximately 136 s) and on phase II (approximately 190 s; Fig. 3A). ESa (10–100 mg/Kg) significantly inhibited the inflammatory phase (phase II) of formalin-induced licking, with a maximal reduction of 69 ± 10% whereas no inhibition was seen in the neurogenic phase (phase I). These results were similar to those obtained with indomethacin, which was ineffective in phase I but inhibited 52 ± 9% of phase II (Fig. 3A).

A maximal effect was already observed with 30 mg/kg ESa in the formalin test. Therefore, this dose was the higher dose chosen for the following experiment. The injection of carrageenan in the hindpaw significantly reduced the mechanical threshold 3 h after the injection (Fig. 3B). Oral treatment with ESa (3–30 mg/kg) dose-dependently inhibited carrageenan-induced mechanical hyperalgesia (Fig. 3B). Indomethacin exerted a similar effect on mechanical hyperalgesia.

### Effects of the ESa in the hot-plate test and motor performance

Oral injection of the ESa 10–100 mg/kg did not change the latency time in the hot-plate test at 55°C compared with vehicle-treated animals whereas fentanyl significantly increased the %MPE compared with vehicle-treated animals (S1 Fig.). Similarly, oral administration of the ESa (10 to 100 mg/kg) did not significantly affect motor behavior in the rotarod test compared with animals that received only the vehicle (S1 Fig.). Conversely, the positive control diazepam significantly reduced motor coordination (79 ± 9%, S1 Fig.).

### Effects of ESa fractions on formalin-induced nociception

Oral treatment with PE and EA fractions at doses of 1.3 mg/kg and 7 mg/Kg, respectively, but not the DM fraction at a dose of 1.5 mg/kg (doses based on yield) obtained from the ESa, significantly inhibited phase II of formalin-induced nociceptive behavior with maximal inhibition of 57 ± 7% for PE and 43 ± 8% for EA with no changes observed in phase I (S2 Fig.). Similar results were obtained for the positive control indomethacin (55 ± 9% reduction in phase II, S2 Fig.). The doses of the fractions were based on the yield considering an effective dose of 30 mg/kg ESa.

### Effect of the isolated compounds on formalin-induced nociception and on carrageenan-induced mechanical hyperalgesia

**Fig 4:**
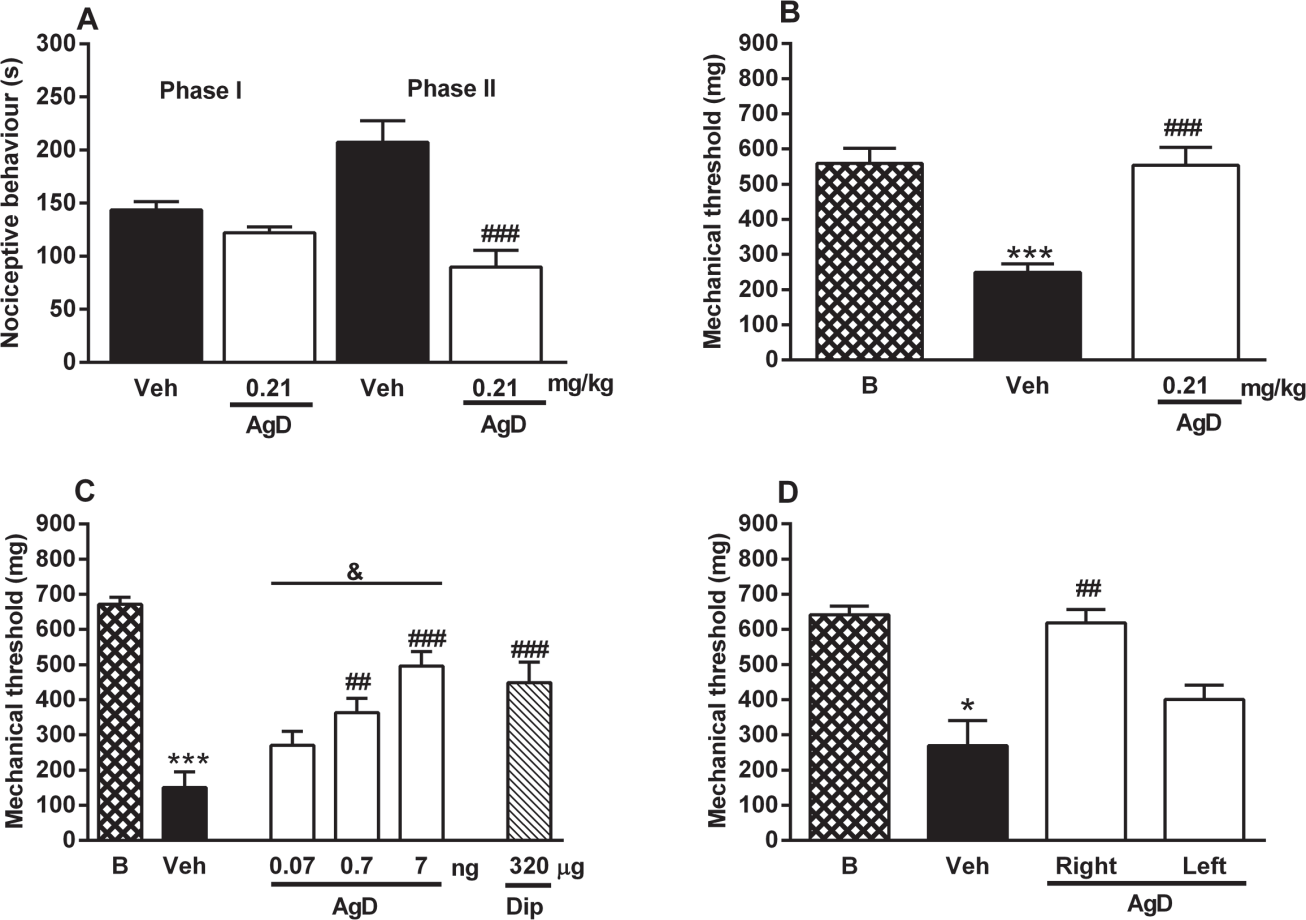
Effect of AgD on nociceptive behavior induced by formalin and on mechanical hyperalgesia induced by carrageenan. Animals were treated with Aggregatin D (AgD) 0.21 mg/kg or vehicle (Veh), by oral route 1 h before the administration of formalin (2.5%, panel A) or carrageenan (Cg, 300 μg, panel B) into the right paw or AgD (0.07, 0.7, 7 ng/paw), Dipyrone (Dip, 320 μg/paw) or vehicle (Veh) 15 min before the injection of Cg (panel C) into the right paw. On panel D, AgD (7ng/paw) was injected in right or left paw as indicated and Cg (300 μg) was injected in the right paw. Basal (B) threshold was evaluated before any injection in panels B, C and D. Formalin-induced nociceptive behavior was evaluated in phase I (0–5 min) or in phase II (15 to 30 min) and the mechanical threshold was evaluated again 3 h after the injection of Cg. Bars represent the mean±s.e.mean of the nociceptive behavior (s) induced by formalin in each phase or the mechanical threshold (n = 10–20). Symbols denote statistical difference in relation to basal threshold (*P<0.05, ***P<0.001) or to veh-treated group (^##^ *P*<0.01, ^###^ *P*<0.001).

Treatment with AgD (0.21 mg/kg, p.o.), a compound isolated from the EA fraction, significantly inhibited the inflammatory phase of formalin-induced licking with maximal inhibition of 56 ± 7% but no significant changes were observed in phase I (Fig. 4A). Treatment with 2-methylanthraquinone and 7-metoxy-2-methylanthraquinone obtained from the PE fraction did not alter the nociceptive behavior induced by formalin (data not shown).

The injection of carrageenan in the hindpaw significantly reduced the mechanical threshold after 3 h (Fig. 4B). Oral treatment with AgD (0.21 mg/kg, dose based on the yield) abolished the carrageenan-induced mechanical hyperalgesia (Fig. 4B).

Local treatment with AgD dose-dependently reduced the carrageenan-induced mechanical hyperalgesia (Fig. 4C) 3 h after the carrageenan injection. AgD (0.7 and 7 ng) significantly reduced mechanical hyperalgesia (41% and 66% inhibiton, respectively), whereas a lower dose (0.07 ng) had no effect. Dipyrone (320 μg) was used as a positive control and had similar effects (57% inhibition, Fig. 4C). Additionally, AgD treatment in the paw contralateral to the carrageenan had no significant effect on mechanical hyperalgesia in contrast to the total inhibition observed with AgD administration in the same paw as the carrageenan (Fig. 4D). The 7 ng dose of AgD was more effective, and this dose was chosen for the subsequent experiments.

### Effect of AgD in the hot plate test and motor performance

Oral administration of AgD (at the same doses that inhibited nociception or a 10-times higher dose) did not change the latency in the hot-plate test at 55°C compared with vehicle-treated animals whereas fentanyl significantly increased the %MPE compared with vehicle-treated animals (S1 Fig.). Similarly, administration of AgD (at the same doses and time points that inhibited nociception and a 10-times higher dose) did not significantly affect motor performance of the animals tested in the rotarod test compared with animals that received only the vehicle (S1 Fig.).

### Effect of AgD on BK-, TNF-α-, IL-1β-, and CINC-1-induced mechanical hyperalgesia

**Fig 5:**
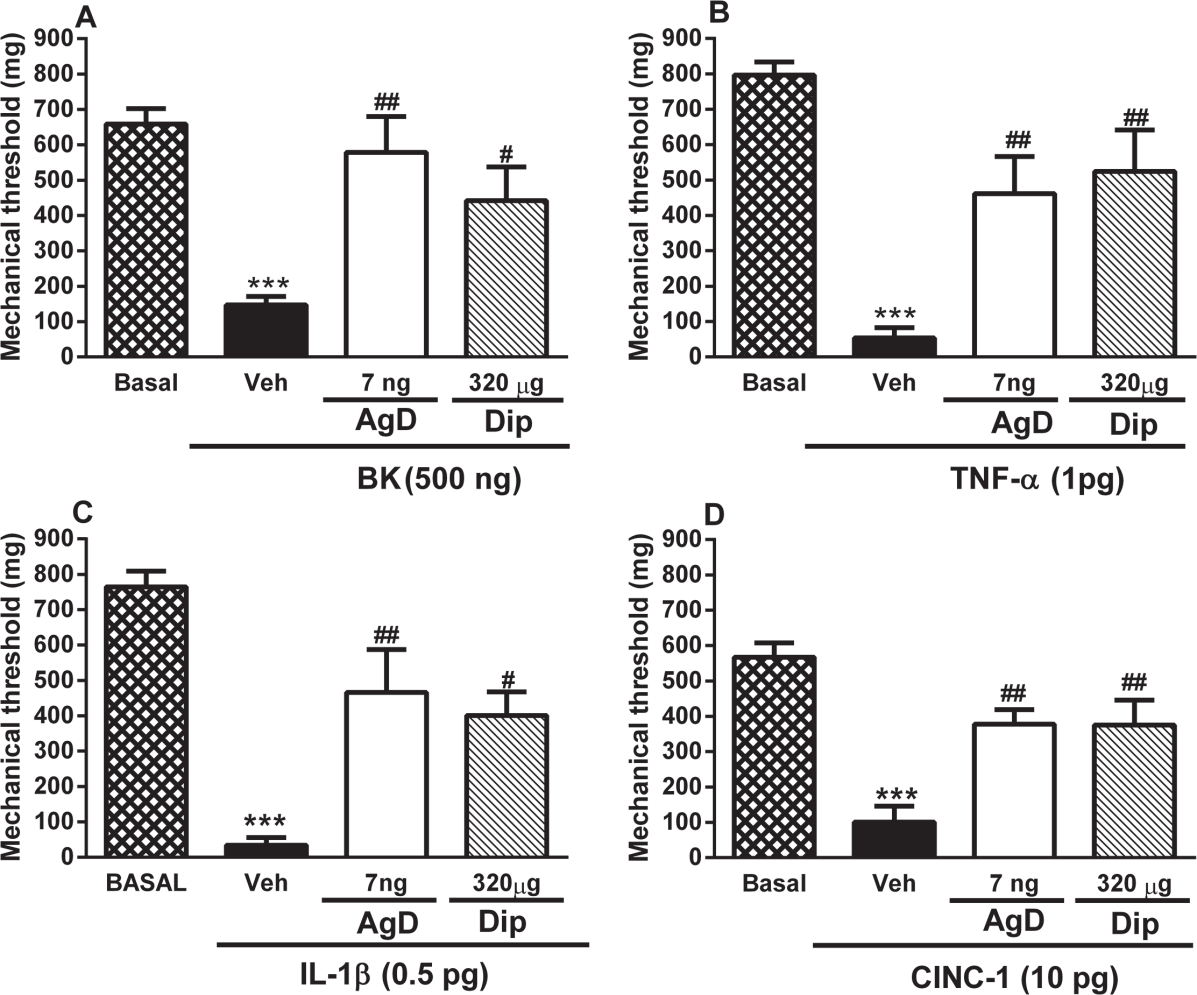
Effect of AgD on mechanical hyperalgesia induced by BK and cytokines. Animals were treated with Aggregatin D (AgD, 7 ng/paw), Dipyrone (Dip, 320 μg/paw) or vehicle (Veh) 15 min before the injection of bradykinin (BK, 500 ng/paw, panel A), tumor necrosis factor-α (TNF-α, 1 pg/paw, panel B), interleukin-1β (IL-1β, 0.5 pg/paw, panel C) or cytokine-induced neutrophil chemoattractant (CINC-1, 10 pg/paw, panel D) in the right paw. Basal (B) threshold was evaluated before any injection. The mechanical threshold was evaluated again 3 h after the injection of the nociceptive stimuli. Bars represent the mean±s.e.mean of the mechanical threshold (mg, n = 8–10). Symbols denote statistical difference in relation to basal threshold (***P<0.001) or to veh-treated group (^#^ *P*<0.05, ^##^ *P*<0.01).

The injection of BK, TNF-α, IL-1β and CINC-1 in the paw significantly reduced the mechanical threshold (Fig. 5A-5D). Local treatment with AgD (7 ng/paw) reversed mechanical hyperalgesia induced by BK and the cytokines by 84%, 55%, 50%, and 59%, respectively. Dipyrone was used as a positive control and also inhibited mechanical hyperalgesia induced by BK and the cytokines by 57%, 63%, 50%, and 59% respectively.

### Effect of AgD on PGE_2_-, dopamine-, forskolin-, and dbcAMP- induced mechanical hyperalgesia

**Fig 6:**
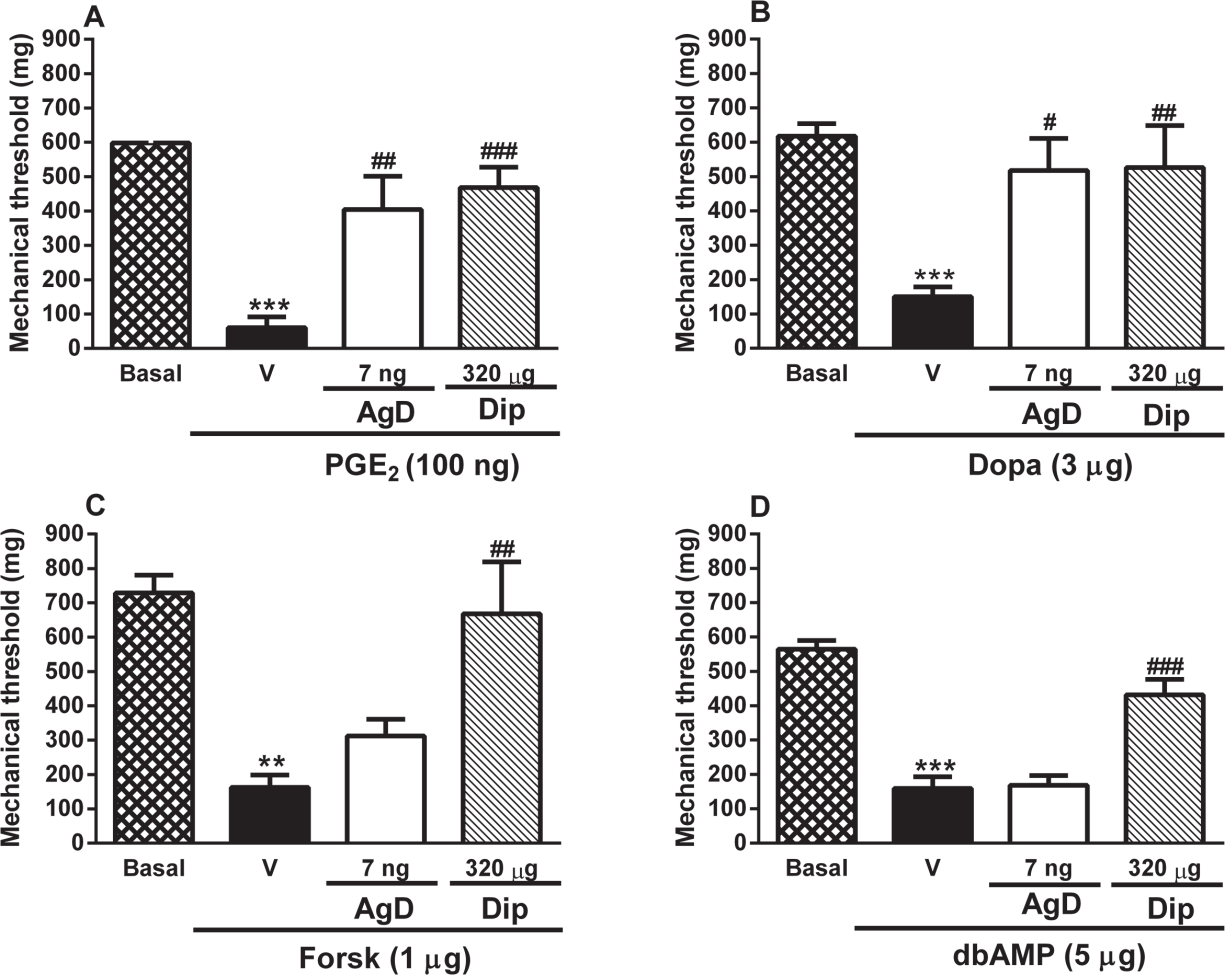
Effect of AgD on mechanical hyperalgesia induced by PGE_2,_ dopamine, forskolin and dbAMPc. Animals were treated with Aggregatin D (AgD, 7 ng/paw), Dipyrone (Dip, 320 μg/paw) or vehicle (Veh) 15 min before the injection of prostaglandin E_2_ (PGE_2_, 100 ng/paw, panel A), Dopamine (Dopa, 3 μg/paw, panel B), forskolin (Forsk, 1 μg/paw, panel C) or dybutiryl cAMP (dbcAMP, 5 μg/paw, panel D) in the right paw. Basal (B) threshold was evaluated before any injection. The mechanical threshold was evaluated again 3 h after the injection of the nociceptive stimuli. Bars represent the mean±s.e.mean of the mechanical threshold (n = 6–16). Symbols denote statistical difference in relation to basal threshold (**P<0.01, ***p<0.001) or to Veh-treated group (^##^ *P*<0.01, ^###^ *P*<0.001).

The injection of PGE_2_, dopamine, forskolin, and dbcAMP in the paw significantly reduced the mechanical threshold (Fig. 6A-6D). Local treatment with AgD (7 ng/paw) reduced the mechanical hyperalgesia induced by PGE_2_ and dopamine by 64% and 78%, respectively (Fig. 6A and 6B). AgD did not alter the reduction of the mechanical threshold induced by forskolin or dbcAMP (Fig. 6C and 6D). Dipyrone was used as a positive control and reduced the mechanical hyperalgesia induced by PGE_2_, dopamine, forskolin and dbcAMP by approximately 76%, 80%, 89%, and 67%, respectively.

### Effect of AgD on NO release

**Fig 7:**
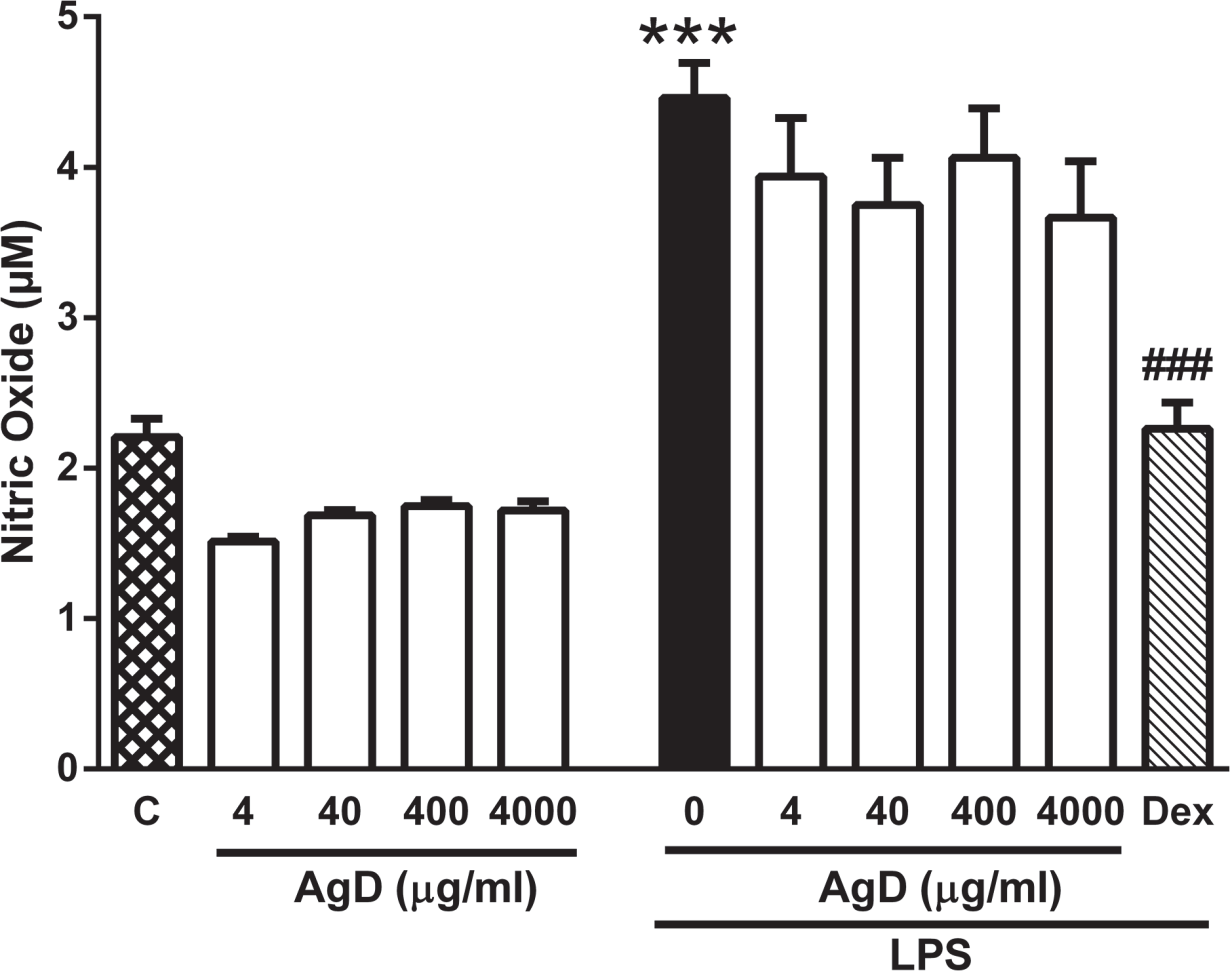
Effect of AgD on NO release by peritoneal macrophages. Mouse peritoneal macrophages (10^6^ cell/well) were treated with Aggregatin D (AgD, 4–4000 ng/ml) or with RPMI 1640 (control, C) in the presence or absence of lipopolysaccharide (LPS, 100 ng/ml). Positive control cells were treated with Dexamethasone (Dex, 9 μg/ml) in the presence of LPS. NO production was quantified through the Nitrite/nitrate production by the Griess method. Bars show the mean±s.e.mean of the nitrite/nitrate (μM) in quadruplicates. Symbols denote statistical difference in relation to control (RPMI) group (***P<0.001) or to LPS-treated group (^###^ *P*<0.001).

The basal production of NO (measured as nitrite/nitrate) by peritoneal macrophages was not significantly changed by treating the cells with AgD, although a small reduction of the NO concentration was observed with all concentrations tested (Fig. 7). LPS significantly increased NO production, but treatment of the cells with AgD did not modify this production (Fig. 7). Conversely, treatment of the cells with the glucocorticoid dexamethasone abolished the increase in NO production induced by LPS (Fig. 7). None of the treatments significantly altered cell viability which was always higher than 95% (data not shown).

### Effect of glibenclamide on the antinociceptive effect of AgD

**Fig 8:**
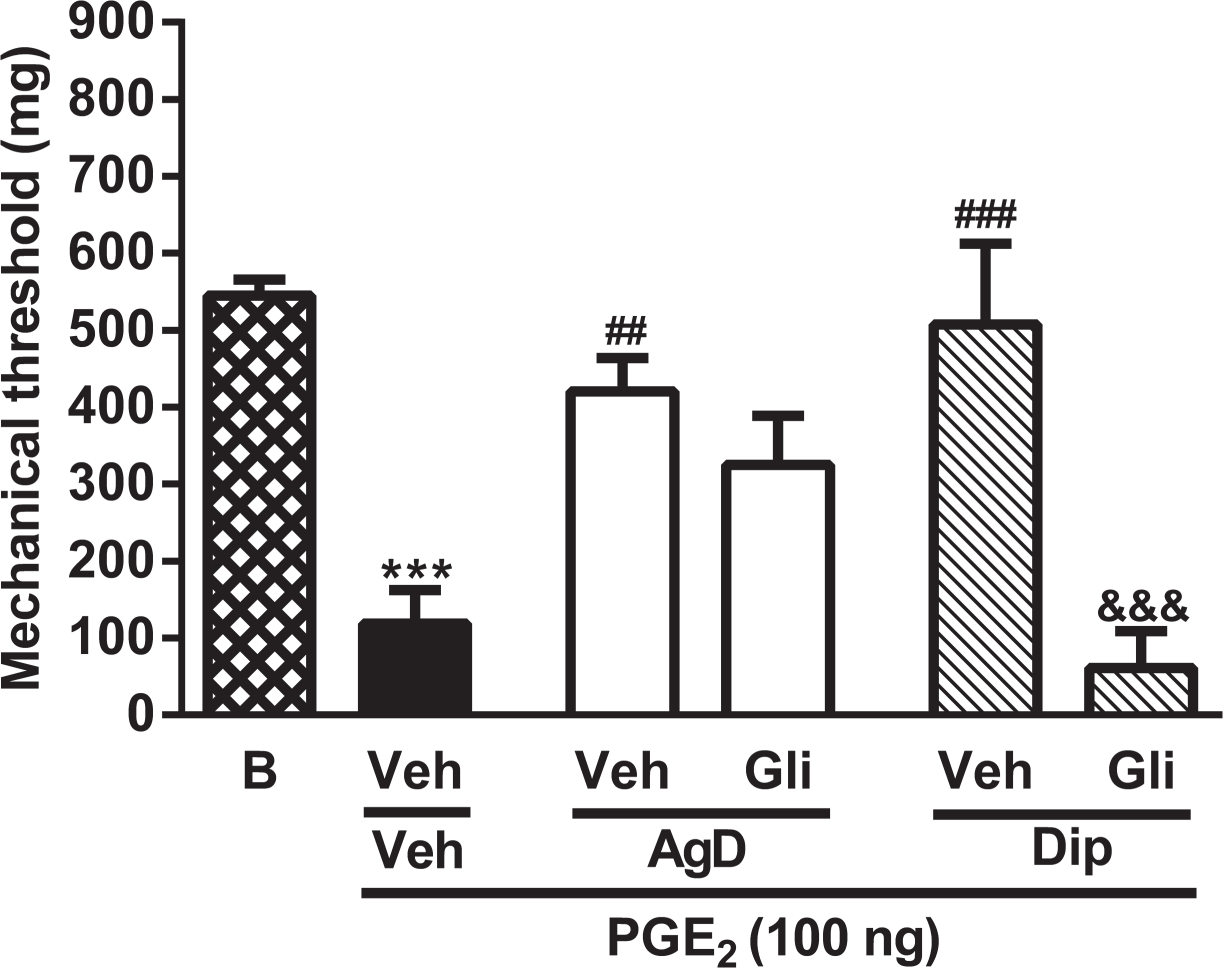
Effect of the potassium channel blocker glibenclamide on the anti-hyperalgesic effect of AgD. Animals were treated with the potassium channel blocker glibenclamide (Gli, 80 μg/paw) or the same volume of vehicle (Veh, Tween 80 1% in saline). After 30 min, animals received Aggregatin D (AgD, 7 ng/paw), Dipyrone (Dip, 320 μg/paw) or vehicle (Veh) followed by an injection of of prostaglandin E_2_ (PGE_2_, 100 ng/paw) 15 min later. Basal (B) threshold was evaluated before any injection. The mechanical threshold was evaluated again 3 h after the injection of the nociceptive stimuli. Bars represent the mean±s.e.mean of the mechanical threshold (n = 5–10). Symbols denote statistical difference in relation to basal threshold (***P<0.001), to Veh/Veh-treated group (^##^ *P*<0.01, ^###^ *P*<0.001) or to the Veh/Dip-treated group (^&&&^ *P*<0.001).

The injection of PGE_2_ reduced the mechanical threshold of the paw by approximately 78% (Fig. 8). Treatment with both AgD and dipyrone completely reversed the mechanical hyperalgesia induced by PGE_2_. Administration of the potassium channel blocker glibenclamide prevented the antinociceptive effect of dipyrone (positive control) but not of AgD (Fig. 8).

### Effect of AgD on nociception induced by capsaicin, cinnamaldehyde, acidic saline and menthol

**Fig 9:**
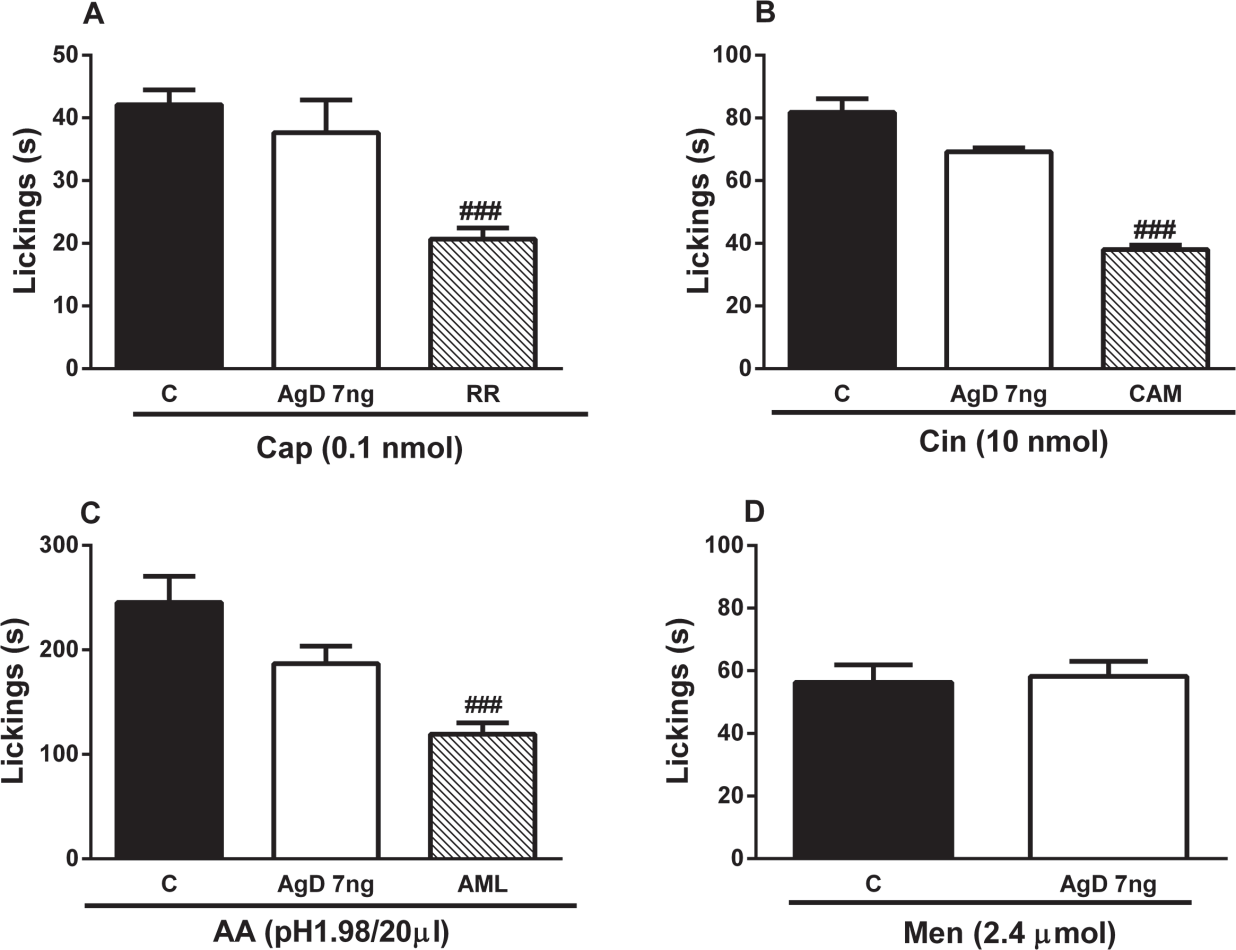
Effect of AgD on nociceptive behavior induced by capsaicin, cinnamaldehyde, acidified saline and menthol. Animals were treated with AgD (7 ng/paw), ruthenium red (RR 1.5 μg/paw), camphor (CAM, 250 ng/paw) or amiloride (AML, 3 μg/paw) or vehicle (Veh) 10 min before the injection of capsaicin (0.1 nmol/paw, panel A), cinnamaldehyde (10 nmol/paw, panel B), acidified saline (pH1.98, 20 μl/paw, panel C) or menthol (2.4 μmol/paw, panel D) in the right paw. The nociceptive behavior was evaluated during 5 min in panels A and B and for 20 min in panels C and D. Bars represent the mean±s.e.mean of the nociceptive behavior (s) (n = 5–8 animals). Symbols denote statistical difference in relation to Veh-treated group (^###^ *P*<0.001).

Capsaicin, cinnamaldehyde, acidic saline and menthol were injected in the hindpaw of the animals inducing a clear nociceptive behavior (Fig. 9) compared with saline-treated animals which did not show any response. Preatrement with these channels blockers ruthenium red, camphor and amiloride significantly decreased the nociceptive behavior induced by these compounds (Fig. 9A-9C). AgD had no effect on nociceptive behavior induced by capsaicin, cinnamaldehyde, menthol, and acidic saline, which are activators of TRPV_1_, TRPA_1_, TRPM_8_, and ASIC_3_ channels (Fig. 9).

## Discussion

The present study showed that the ethanolic extract obtained from the tubers of *S*. *aggregata* has important antinociceptive activity, in which it blocked the inflammatory phase of overt nociception induced by formalin and mechanical hyperalgesia induced by carrageenan. However, the ESa did not exert an antiinflammatory or antipyretic effect. A new compound identified in this plant, AgD, shared this antinociceptive activity, likely acting at peripheral sites. Additionally, AgD blocked mechanical hyperalgesia induced by BK, TNF-α, IL-1β, CINC-1, PGE_2_ and dopamine but not forskolin, dbcAMP, capsaicin, cinnamaldehyde, menthol, or acidic saline. The analgesic effect of AgD also did not appear to involve the NO/cGMP/K^+^ channel pathway.

The ESa was effective against inflammatory nociception (formalin-induced and mechanical hyperalgesia). However, ESa, at least at the doses tested, does not act similarly to NSAIDs or glucocorticoids, because these drugs effectively inhibit edema formation [43], neutrophil migration [44] and fever [26, 45]. The ESa also did not appear to act similarly to opiods, another class of commonly used analgesic drugs, because opioids effectively block the first phase of formalin-induced nociceptive behavior in mice [29] and physiological heat-induced nociception (S1 Fig.). Although dipyrone and acetaminophen are two NSAIDs that are known to have weak anti-inflammatory action [46, 47] they exert antipyretic effects at similar or lower doses than those required for their analgesic effects [48, 49]. Therefore, these data suggest that the ESa contain compounds that mainly have antinociceptive effects that act specifically on nociceptor sensitization.

After fractioning, two of these ESa-derived fractions showed a similar antinociceptive activity: the PE fraction and EA fraction. From the PE fraction two anthraquinones, 2-methylanthraquinone and 7-methoxy-2-methylanthraquinone, were isolated. Anthraquinones are potential antinociceptive drugs. For example, the anthraquinones diacerhein and emodin possess antinociceptive activity in different models [50, 51]. Nevertheless, both anthraquinones that were isolated from the PE fraction failed to change formalin-induced nociception at the doses tested. Higher doses were not tested but these anthraquinones are unlikely responsible for the antinociceptive activity of the ESa because the doses used were the same as the PE fraction. Sitosterol, a compound found in the PE fraction, possesses antinociceptive activity at doses that are 10 times higher than the one used in the present study [52], suggesting that it is not responsible for the activity of this fraction. Therefore, compounds other than those isolated in the present study may be responsible for the antinociceptive activity of the PE fraction. This will be an issue for future studies.

Conversely, a new hydronaphthoquinone derivative identified in the EA fraction, named AgD, very effectively reduced both carrageenan-induced mechanical hyperalgesia and the formalin-induced nociception. These data suggest that the antinociceptive activity of the ESa is at least partially related to the presence of AgD. Notably, the doses of the ESa and AgD that completely abolished carrageenan-induced hyperalgesia only partially reduced the nociceptive behavior induced by formalin. This may be attributable to the fact that formalin evokes very complex behavior that involves the sensitization and/or activation of nociceptors, and AgD specifically reduces nociceptor sensitization. Additionally, the ESa and AgD failed to produce antinociceptive effects when a thermal stimulus (i.e., the hot plate) was used thus substantiating their preferential peripheral action in nociceptive responses of inflammatory origin. We also evaluated the mice in the rota rod test, and confirmed that neither the ESa nor AgD affected motor performance at the doses tested which could otherwise affect the results of the nociception tests. AgD did not affect the motor performance even at a dose that was 10 times higher than the antinociceptive dose.

AgD also effectively reduced mechanical hyperalgesia when administered locally, suggesting that it acts directly at the inflammatory site. The local effect was confirmed when the same dose of AgD that was injected in the contralateral paw exerted no antinociceptive effect. Carrageenan-induced mechanical hyperalgesia in mice depends on the release of BK, TNF-α, IL-1β, and KC and subsequently of sympathetic amines and prostaglandins [53–55]. Based on this sequence of mediators, we attempted to unravel the mechanism of action of AgD. Our results clearly showed that AgD prevented sensitization induced by BK, TNF-α, IL-1β, and CINC-1, a chemokine that shares the same receptor with KC [56]. These results suggest that AgD was not blocking the synthesis or release of these mediators and acted after their release at the inflammatory site.

However, AgD also significantly reduced mechanical hyperalgesia induced by both PGE_2_ and dopamine, which are described as the final mediators of inflammatory hyperalgesia [4, 53, 54]. PGE_2_ biosynthesis depends on the conversion of membrane arachidonic acid by the action of cyclooxygenase [11]. Therefore, the blockade of PGE_2_-induced hyperalgesia suggests that AgD does not act as an NSAID by inhibiting cyclooxygenase-2 activity. Once released, PGE_2_ interacts with G protein-coupled receptors subtypes EP_2_ and/or EP_4_, which are expressed in peripheral sensory neurons. The activation of these receptors results in the activation of adenylyl cyclase and increase in the levels of cAMP, which directly promotes the activation of protein kinase A. Protein kinase A, in turn, has actions on various ion channels that sensitize nociceptors [57, 58]. Alternatively, sympathetic amines have been shown to be involved in the development of hyperalgesia by functionally upregulating nociceptors [59, 60]. Dopamine is a sympathetic amine that is released during inflammation and promotes the direct sensitization of nociceptive neurons in a manner that depends on the activation of dopamine D1 receptors that stimulate the adenylyl cyclase/cAMP pathway [61–64].

Therefore our next step was to evaluate wether AgD reverses mechanical hyperalgesia by acting on these intracellular components. Mechanical hyperalgesia was induced by the adenylyl cyclase activator forskolin and dbcAMP, a permeable analogue of cAMP which is a direct activator of protein kinase A. Both, forskolin and dbcAMP reduced the paw withdrawal threshold to the same extent as PGE_2_ and dopamine. However, at the dose tested, AgD did not reverse mechanical hyperalgesia induced by these agents. As expected, the positive control dipyrone reduced the mechanical hyperalgesia induced by all of the stimuli [4, 46, 65]. These results suggest that AgD possess a mechanism of action different from dipyrone and has actions that occur between the activation of G-protein-coupled receptors and activation of the adenylyl-cyclase/cAMP pathway.

Some studies have shown that naphthoquinones might have antinociceptive/antiinflammatory activity through the inhibition of NF-κB activity. Ahn et al. showed that a furonaphthoquinone compound suppressed cyclooxygenase-2 expression in RAW 264.7 macrophages which may confer potential antiinflammatory activity to this compound [66]. Similarly, Song et al. showed that isoeleutherin suppressed the expression of inducible NO synthase and various cytokines by inhibiting NF-κB activity [67]. The effectiveness of AgD on PGE_2-_induced hyperalgesia suggests that it does not act by inhibiting NF-κB activity. To further support this hypothesis, we found that treating macrophages with AgD did not change the LPS-induced NO production at any of the concentrations tested. Therefore, AgD did not appear to exert a similar effect as the one observed for isoeleutherin [67].

Dipyrone has a specific antinociceptive effect on PGE_2_-induced hyperalgesia, which is not shared by most of the cyclooxygenase inhibitors, such as indomethacin. Lorenzetti & Ferreira and Duarte et al. showed that the peripheral effect of dipyrone was mediated by the activation of the _L_-arginine/NO/cGMP pathway [46, 65]. Subsequently, Alves & Duarte (2002) demonstrated that the antinociceptive effect of dipyrone in PGE_2_ induced mechanical hyperalgesia could be completely reversed by the local application of glibenclamide, an adenosine triphosphate-sensitive potassium channel blocker. In sharp contrast to dipyrone, the antinociceptive action of AgD was not reversed by glibenclamide, confirming that AgD does not act through this pathway.

The reduction of the paw withdrawal threshold induced by BK in mice may occur independently of cytokine release [2]. This peptide may cause nociception through its ability to directly activate nociceptors [68] by inducing the release of prostanoids and sympathetic amines [54, 61, 69] or binding to its G protein-coupled receptor B_2_ which is constitutively expressed in nociceptive fibers and promotes the activation of protein Gq/11, followed by the release of inositol triphosphate and diacylglycerol [68, 70]. The latter is responsible for nociceptive behavior by promoting the activation of protein kinase C, which can phosphorylate various ion channels [68, 71, 72]. Because AgD effectively reduced nociception induced by BK, we decided to test wether this compound acts on ion channels.

Transient receptor potential channels, one of the largest families of ion channels, are considered signal transducers that may participate in nociception [73]. We observed that AgD was ineffective in inhibiting nociceptive behavior induced by the TRPV_1_ channel activator capsaicin, TRPA_1_ channel activator cinnamaldehyde, ASIC_3_ channel activator acidic saline, and TRPM_8_ channel activator menthol [73]. Conversely, the nociceptive response produced by capsaicin was significantly reduced by ruthenium red, an inorganic polycationic dye that nonselectively blocks the response to several TRP channels. Additionally, the nociceptive responses induced by cinnamaldehyde and acidic saline were blocked by camphor and amiloride that are TRPA_1_ and ASIC antagonists, respectively.

In conclusion, the Esa had antinociceptive activity at doses that were not anti-pyretic or antiinflammatory. This antinociceptive activity was related, at least partially, to the presence of AgD, a new compound identified in this *Sinningia* species. AgD, at nanomolar doses signficantly reduced inflammatory pain by primarily preventing the sensitizing actions of the major mediators of inflammatory pain, such as cytokines, BK, PGE_2_, and dopamine. Its antinociceptive effect appeared to be peripheral and different from classic NSAIDs, glucocorticoids, opioids and dipyrone. AgD may interfere in some step between G-protein-coupled receptor activation and cAMP formation.

## Supporting Information

S1 Fig Effect of ESa or AgD on motor performance and hot-plate test in mice. Animals were treated with ethanolic extract form *S*. *aggregata* (ESa, 10, 30 and 100 mg/kg, as indicated), aggregatin D (AgD, 0.21 or 2.1 mg/kg) or the appropriate vehicles (Veh) by oral route or subcutaneously with diazepam (Dzp, 5 mg/kg) or fentanyl (Fent, 0.5 min). One hour after ESA or AgD or 15 min after Dzp or Fent animals were submitted to the rota-rod task (panel A) or hot-plate test (panel B). Data show the mean ± s.e.mean of the time spent in the rota-rod or the % of the maximal possible effect on the hot-plate test (n = 6–12). Symbols denote statistical difference in relation to Veh-treated group (***P<0.01).
(TIF)

S2 Fig Effect of ESa fractions on nociceptive behavior induced by formalin. Animals were treated with fractions petroleum ether (PE, 1.3 mg/kg), dichoromethane (DM, 1.5 mg/kg), ethyl acetate (EA, 7 mg/kg), indomethacin (Ind, 5 mg/kg) or vehicle (Veh), by oral route 1 h before the administration of formalin (2.5%, panel A) into the right paw Formalin-induced nociceptive behavior was evaluated in phase I (0–5 min) or in phase II (15 to 30 min). Bars represent the mean±s.e.mean of the nociceptive behavior (s) induced by formalin in each phase (n = 10–12). Symbols denote statistical difference in relation to veh-treated group (^#^ *P*<0.05, ^##^ *P*<0.01).
(TIF)
