# Supplementary figures and images for: Antinociceptive Activity of the Ethanolic Extract, Fractions, and Aggregatin D Isolated from Sinningia aggregata Tubers
Source: PLoS One. 2015 Feb 26;10(2):e0117501. doi: 10.1371/journal.pone.0117501 (PMC4342217; doi:10.1371/journal.pone.0117501)

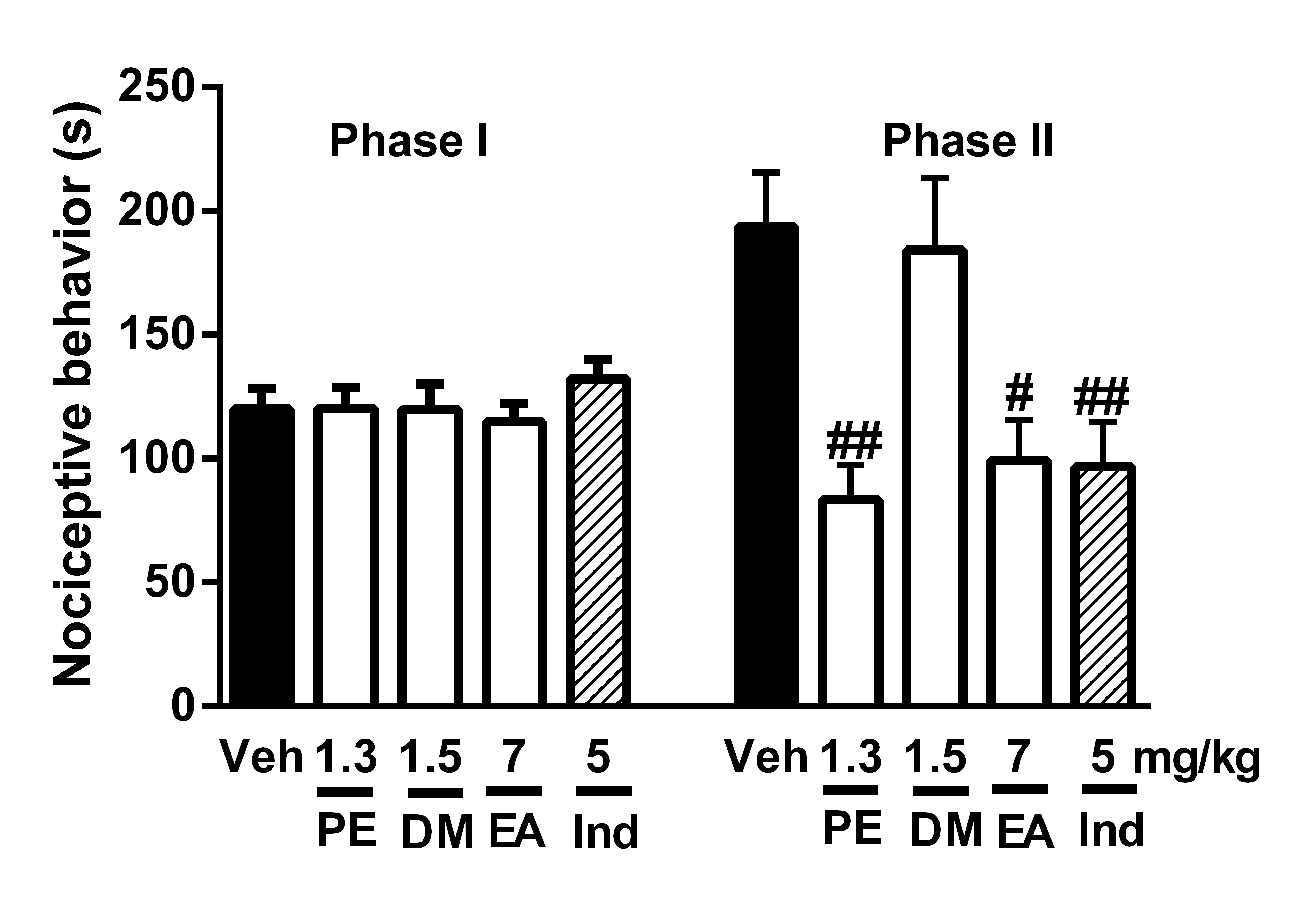

Supplement: S2 Fig — Animals were treated with fractions petroleum ether (PE, 1.3 mg/kg), dichoromethane (DM, 1.5 mg/kg), ethyl acetate (EA, 7 mg/kg), indomethacin (Ind, 5 mg/kg) or vehicle (Veh), by oral route 1 h before the administration of formalin (2.5%, panel A) into the right paw Formalin-induced nociceptive behavior was evaluated in phase I (0–5 min) or in phase II (15 to 30 min). Bars represent the mean±s.e.mean of the nociceptive behavior (s) induced by formalin in each phase (n = 10–12). Symbols denote statistical difference in relation to veh-treated group (# P<0.05, ## P<0.01). (TIF) [file pone.0117501.s002.tif]
